# MCM-test: a fuzzy-set-theory-based approach to differential analysis of gene pathways

**DOI:** 10.1186/1471-2105-9-S6-S16

**Published:** 2008-05-28

**Authors:** Lily R Liang, Vinay Mandal, Yi Lu, Deepak Kumar

**Affiliations:** 1 Department of Computer Science and Information Technology, University of the District of Columbia, Washington, D.C., USA; 2 Department of Computer Science, Wayne State University, Michigan, USA; 3 Department of Computer Science, Prairie View A&M University, Texas, USA; 4 Department of Biological and Environmental Sciences, University of the District of Columbia, Washington, D.C., USA

## Abstract

**Background:**

Gene pathway can be defined as a group of genes that interact with each other to perform some biological processes. Along with the efforts to identify the individual genes that play vital roles in a particular disease, there is a growing interest in identifying the roles of gene pathways in such diseases.

**Results:**

This paper proposes an innovative fuzzy-set-theory-based approach, Multi-dimensional Cluster Misclassification test (MCM-test), to measure the significance of gene pathways in a particular disease. Experiments have been conducted on both synthetic data and real world data. Results on published diabetes gene expression dataset and a list of predefined pathways from KEGG identified OXPHOS pathway involved in oxidative phosphorylation in mitochondria and other mitochondrial related pathways to be deregulated in diabetes patients. Our results support the previously supported notion that mitochondrial dysfunction is an important event in insulin resistance and type-2 diabetes.

**Conclusion:**

Our experiments results suggest that MCM-test can be successfully used in pathway level differential analysis of gene expression datasets. This approach also provides a new solution to the general problem of measuring the difference between two groups of data, which is one of the most essential problems in most areas of research.

## Background

Current microarray technologies conduct simultaneous studies of gene expression data of thousands of genes under different conditions. In most of these studies, expression data are analyzed using various standard statistical methods to identify a list of genes responsible for a particular condition. However, in these approaches, interplay among genes is not taken into account. Since organisms behave as complex systems, functioning through chemical networks and interaction of various molecules (also known as pathways), this interplay of genes may provide invaluable insight to the understanding of various diseases. Thus, along with the efforts to identify the individual genes that play vital roles in a particular disease, there is a growing interest in identifying the roles of different pathways in such diseases.

Biological pathway is a group of related genes coding for proteins that interact with each other to perform some biological processes. According to the biological processes they are involved with, pathways can be divided into several categories, such as metabolic pathways and regulatory pathways. Metabolic pathways are series of chemical reactions occurring within a cell, catalyzed by enzymes, resulting in either the formation of a metabolic product to be used or stored by the cell, or the initiation of another metabolic pathway. Regulatory pathways represent protein-protein interactions.

During the past few years, many signaling and metabolic pathways have been discovered experimentally and have been integrated into pathway databases, such as KEGG [1] and Biocarta [2]. Various statistical techniques have been developed to analyze microarray expression data for the relevance of predefined pathways to a particular disease. These techniques include gene set enrichment analysis [3,4], pathway level analysis of gene expression using singular value decomposition by Tomfohr et al. [5], and hypothesis testing [6] by Tian et al. These approaches are reviewed in detail in the related works section.

Generally speaking, these approaches can be divided into two categories:

• Conduct statistical differential analysis at the individual gene level, and integrate the result statistics of the genes in the same pathway;

• Obtain activity level indices of each pathway for different sample groups and conduct differential analysis of these indices.

For the first category, when the statistics at individual gene level miss significant genes, the effectiveness of the pathway analysis will be affected. An example is given in the later part of this section. For the second approach, extracting pathway activity level indices from gene expression data may cause loss of information.

Diabetes is a group of diseases characterized by high levels of blood glucose resulting from defects in insulin production, insulin action, or both. It is one of the most common diseases, affecting 18.2 million people in the United States, or 6.3% of the population [7]. Hence, identifying active pathways in diabetes is a critical task for understanding this disease. Several pathway analysis works have been proposed in this area [3,5,6].

In gene set enrichment analysis (GSEA) [3], a differential statistic is calculated first for each gene from their expression data of two different groups of samples. Then the genes are ordered according to the statistic values. A running sum of weights is calculated from the ordered list for a particular pathway. The maximum value of this running sum is called the enrichment score of that pathway. To measure the significance of this score, a null distribution of enrichment scores is generated by permuting the sample labels. This approach falls into the first category stated previously, i.e., statistical analysis at individual gene level is performed followed by an integration of these statistics of genes in the same pathway.

In [5], a hypothesis testing framework for pathway differential analysis is proposed. T-test and Wilcoxon rank test are recommended to measure the difference of expressions of a single gene between two groups of samples. Then this statistic is accumulated over each gene in a particular pathway and standardized by the total number of genes in this pathway. The significance of the result is then interpreted by rejecting two null hypotheses, each with a null population generated by permuting sample labels or gene indices. This approach also belongs to the first category above. Statistical analysis at individual gene level is still required for the pathway analysis in this approach.

In [6], singular value decomposition is used to obtain pathway activity levels from the gene expression matrix. T-test is applied to the pathway activity levels of the two different sample groups to measure the difference. Significance of the measurement is also obtained by permuting the sample labels. In this approach, no differential analysis at individual gene level is required. However, an extraction of pathway activity level prior to the differential analysis is required. During this extraction process, since only the first eigenvector of singular value decomposition is used, some information of expressions is lost. This approach belongs to the second category stated above.

As discussed above, either t-test or rank sum test is used as a core step by [3,6] to identify individual genes which are expressed differently from two different sample groups. Thus these methods inevitably inherit the disadvantage of t-test and rank sum test. While the t-test is very sensitive to extreme values and cannot distinguish two sets with close means even though the two sets are significantly different from each others, the rank sum test is not sensitive to absolute values. In turn, those pathways contain genes which can not be identified by t-test or rank sum test but actually are significantly differently expressed in two different sample groups will be affected. For example, as showed in Table 1, the expressions of Gene 3 are significantly different under two conditions. However this gene was not identified by t-test. Thus, a pathway involving this gene is less likely to be identified by the first category of analysis that uses t-test at the gene level.

**Table 1:** An example of five gene pathway

Gene ID	*S*_1_					*S*_2_					CM *d*-value	*P*-value		
												
												CM-test	t-test	Rank Sum test
1	750	559	649	685	636	310	359	135	97	178	1	0.001	0.000	0.008
2	391	379	268	323	380	774	506	416	468	449	1	0.005	0.029	0.008
3	598	424	695	451	141	342	260	266	229	234	0.904	0.018	0.077	0.152
4	233	216	193	394	327	436	980	363	424	416	0.905	0.017	0.071	0.015
5	305	221	241	183	158	201	176	189	177	250	0.812	0.143	0.448	0.693

In this paper, we propose an innovative fuzzy-set-theory-based approach for differential analysis of gene pathways and apply it on identifying significant pathways for diabetes. In our proposed MCM-test, instead of identifying individual genes first, the differential analysis is done directly at the pathway level without individual gene differential statistic. All expression values of genes which belong to a pathway of a particular patient are treated as a vector. The intuition behind this is based on the fact that genes for each patient interplay with each other. MCM-test does not extract activity level of pathways either. This allows keeping the maximum amount of information for the pathway differential analysis. Moreover, the fuzzy concept makes the approach more tolerant to individual data item noise.

## Results

To investigate our approach, we conducted experiments on both synthetic data and real world data. We first conducted a series of experiments on synthetic datasets to find the characteristics of MCM *d*-value. We then used the MCM-test on the real world diabetes dataset analyzed by Tomfohr et al. [5] and GSEA [3]. Results on real world diabetes data identified several pathways that were deregulated in diabetes patients. The top three pathways identified were related to mitochondrial functions in accordance with previous diabetes studies. Mitochondrial dysfunction is known to be related to insulin resistance and type-2 diabetes. Our data suggests that the method can be successfully used in pathway level differential analysis of gene expression datasets.

### Relationship between MCM d-value and mean difference of the distributions

Suppose two sets S_1 _and S_2 _are drawn from two different distributions, then a good divergence value will satisfy the following property: the less the overlap, the higher the d-value. To validate that our MCM-test has this property, we performed the following steps:

1. generated 17 values from Gaussian distribution N (*μ*, *σ*), where *μ *is the mean and *σ *is the variance, to use as gene expression data. The number 17 was chosen to mimic the real world diabetes dataset used for the analysis in this paper.

2. repeated Step 1 for 100 times to get expression data of 100 genes

3. generated 17 values from Gaussian distribution N (*μ *+ x, *σ*), with x = 0 at this time.

4. repeated Step 3 for 100 times

5. analyzed these 100 pairs of sets of values with MCM-test and obtained the d-value.

6. repeated Step 1 to Step 5 for 1000 times and averaged the d-values over all the iterations.

7. repeated Step 1 to Step 6 for each x: x = 0, 20, 40, 60, 80, 100, 120, 140, 160, and 180.

**Figure 1:**
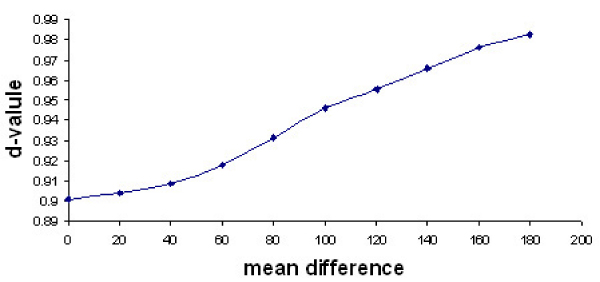
**Relationship between d-value and mean difference**. Two datasets are generated from two distributions *N *(*μ*, *σ*) and *N *(*μ *+ *x*, *σ*). As the mean difference, *x*, increases, the d-value also increases.

Figure 1 shows the average d-value verses mean difference. We can see that the MCM-test has the desired property: the larger the mean difference between two sets, the larger the divergence d-value.

### Impact of population size on standard deviation of MCM d-value

**Figure 2:**
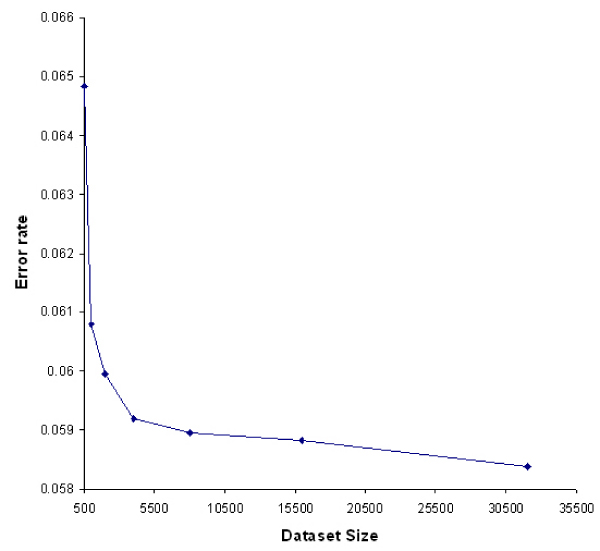
**Impact of number of permutation on the error rate of PDF of MCM d-value**. We show the error rate for various numbers of permutations ranging from 500 to 32000. The error rate decreases as the number of permutations increases.

At this point, a natural question is, what the standard deviation of MCM d-values look like and how population size of d-value influences it. To answer these questions, we generated sample expression datasets and calculate d-values following a process similar to step 1 to 5 in the previous section. Again, we fixed the pathway length to 100. We repeat the process and obtained 500 d-values and calculated the standard deviation of the d-values. This is then repeated for 10 times and the 10 standard deviations are averaged and recorded as error rate of MCM d-value for that population size 500. Similarly we obtained the error rate for various d-value population sizes. As shown in Figure 2, the error rate decreases as the dataset size increases. We also note that the error rate becomes stable after the size of the population becomes greater than 8000.

### Relationship between MCM d-value and empirical p-value

**Figure 3:**
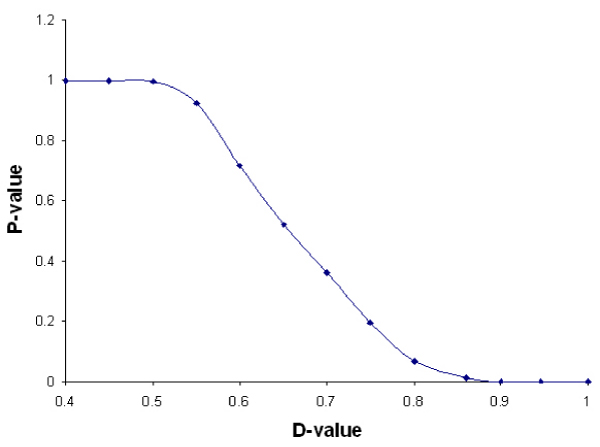
**Relationship between MCM d-value and its empirical p-value**. As the d-value increases, the corresponding empirical p-value decreases.

Suppose two vectors S_1 _and S_2 _are drawn from same Normal distribution. What is the probability that the MCM d-value of these vectors is greater than a particular D? Does the probability increases with the increase of D? To answer these questions, we studied the relationship between MCM d-value and empirical p-value as follows:

1. We generated 15000 pairs of sets, each set with 15 values from standard normal distribution.

2. From these 15000 pairs of sets, we randomly selected 100 pairs of sets to simulate expression data of a pathway with 100 genes under two conditions. We calculated d-value for this pathway. Since we know that the data size required to obtain stable standard deviation of d-value is 8000 from the previous experiment, this process is repeated 10000 times.

3. For each pathway generated above with d-value *D*, we calculated the empirical p-value as *n*+1/10001, where n is the number of d-values generated above that are equal to or greater than *D*. The relationship between the d-value and p-value is shown in Figure 3.

In Figure 3 we can see that as the d-value increases, the p-value decreases. In particular, when d-value is greater than 0.809, we have p-value ≤ 0.05.

### Impact of number of samples on error rate of MCM-test d-value

**Figure 4:**
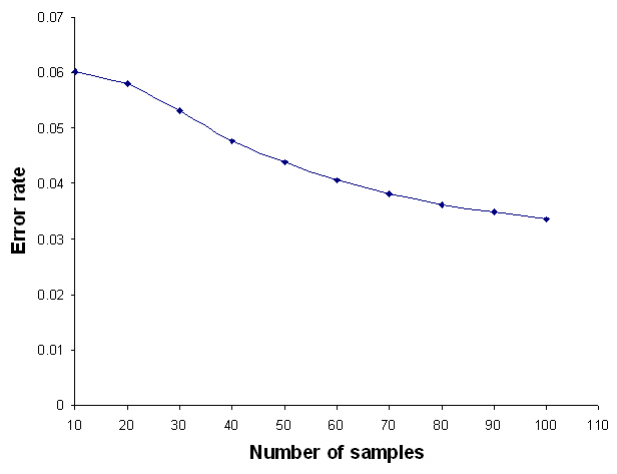
**Impact of number of samples on error rate of PDF of MCM-test d-value**. As the number of samples in a pathway increases, the error rate of PDF of MCM d-value also decreases.

In order to understand the effect of the number of samples on error rate of MCM d-value, we generated datasets with different sample sizes. For each sample size, we generated 10000 datasets and calculated the corresponding 10000 d-values. The standard deviation of these d-values was calculated. This process is repeated 10 times and the average of the standard deviations is recorded as the error rate. The same is done for the other sample sizes. The relationship between number of samples and the error rate is shown in Figure 4. As expected, the error rate decreases as the number of samples increases.

### Analyzing the diabetes dataset with MCM-test

The diabetes dataset contains the transcriptional profiles of smooth muscle biopsies of diabetic and normal individuals. In the expression dataset, for each gene, there are 17 expression values from subjects with type 2 diabetes (DM2), 17 expression values from subjects with normal glucose tolerance (NGT) and 10 expression values from subjects with impaired glucose tolerance (IGT). For our analysis, we only used the 34 expression values from subjects with type 2 diabetes and subjects with normal glucose tolerance. Furthermore, we used about 150 pathways obtained from KEGG (Kyoto Encyclopedia of Genes and Genomes) [1].

The expression values in the dataset which are too small, i.e., less than 100 are considered to be the result of noise. So, to minimize the effect of these low values, we only included the genes which have at least one of the expression values greater than 100. Out of the 22,283 genes in the dataset, 10,983 met the filtering criteria. The d-value for each pathway was calculated as described in the methodology section before. The p-value for the pathway was calculated using permutation test. We permuted the genes 1000 times, each time selecting the same number of genes as that of the pathway under consideration. We then calculated the d-value of each pathway obtained thus and the p-value for the pathway was the fraction of times the d-values of the pathways obtained by 1000 permutation equaled or exceeded the original d-value.

The pathways are ordered in the ascending order of their p-values. The significant pathways, i.e., the pathways with p-value less than 0.05, are then ordered according to the percentage of the genes in the pathway which were represented in the dataset. Table 2 shows the result after sorting.

**Table 2:** The results from MCM-test on diabetes dataset

Pathway Name	MCM-test p-value	No. of genes hits in dataset	Actual no. of genes in pathway	Percentage of gene hits
OXPHOS	0.04995	106	114	92.98
c20 U133 probes	0.013	215	270	79.62
human mitoDB	0.029	436	594	73.4
c33 U133 probes	0.021	245	362	67.67
MAP00252	0.022	23	35	65.71
c34 U133	0.012	274	452	60.62
c21 U133	0.026	166	287	57.84
c8 U133	0.013	164	288	56.94

Using our method, we identified the deregulation of mitochondrial pathways in the dataset which is in accordance with previous studies. The first cluster of genes involved was from the mitochondrial OXPHOS pathway. The OXPHOS pathway was well represented in the data with 93% of genes (106 out of 114) present in the dataset. Oxidative phosphorylation in mitochondria provides energy in the form of ATP generation. In muscle cells, mitochondrial dysfunction has been linked to insulin resistance and type-2 diabetes [8-10]. The involvement of genes coded by mitochondria in insulin resistance is also well known. The depletion of cellular mitochondrial DNA has been shown to cause insulin resistance in experimental model [11]. Reduced mitochondrial oxidative phosphorylation leads to the accumulation of intracellular triglycerides resulting in insulin resistance. The next 2 clusters, c20_U133 which is a manually curated cluster of genes coregulated with OXPHOS [3] and the mitochondrial gene cluster human_mitoDB_6_2002 reinforce that muscle mitochondrial dysfunction is linked to type-2 diabetes.

## Conclusion

In this paper, we propose an innovative fuzzy-set-theory-based approach for differential analysis of gene pathways and apply it on identifying significant pathways for diabetes. Experiments have been conducted on both synthetic datasets and real world dataset. Results on real world diabetes data identified several number of gene pathways. Of note our top significant pathways were related to mitochondrial function which is well known to be involved in insulin resistance and type-2 diabetes. This approach can be used not only for pathway analysis of other diseases but also for other domains. As measuring the difference of two groups of data are essential to most of researches, our approach provides a solution to this general and most critical problem.

## Methods

In [12-14], we proposed two fuzzy-set-theory based methods, CM-test and FM-test, to identify the individual genes that expressed significant differences under two conditions. In this paper, we extended the cluster misclassification concept to a multi-dimensional space and propose a new approach for pathway analysis, Multi-dimensional Cluster Misclassification test (MCM-test). Comparing with CM-test and FM-test, MCM-test looks for a group of genes significant under two conditions instead of identifying significant individual genes under two conditions. In this approach, the expression values of a group of *Q *genes for a particular sample under a particular condition are considered as a *Q*-dimension vector. The differential analysis is done at the vector level, without individual gene differential statistic.

In this section, we first introduce the concept of fuzzy membership function of vectors, then the details of MCM-test.

### Fuzzy membership Function of Vectors

**Figure 5:**
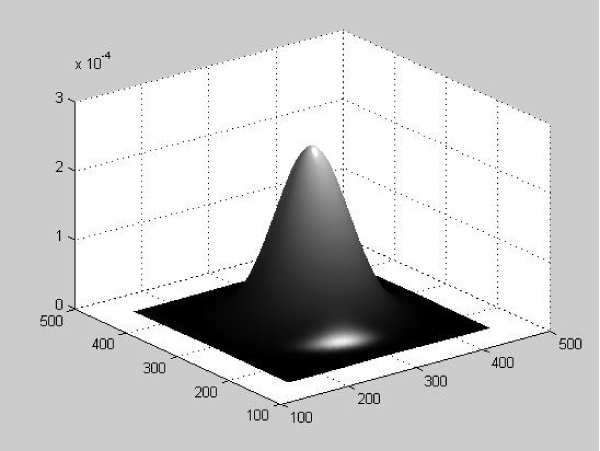
A sample fuzzy membership function of vector (x, y).

In fuzzy set theory, the degree for one variable to belong to a fuzzy set is defined by a function. For a vector which has two dimensions, the degree that it belongs to a set of vectors can be defined by a three-dimensional function, with the third dimension being the measurement of the membership. Figure 5 shows a sample fuzzy membership function for a vector (*x*, *y*).

For vectors with *n *dimensions, their fuzzy membership function will be *n+1*-dimensional, with one dimension measuring the fuzzy membership.

### Our approach

Consider a pathway that consists of *Q *genes, the problem now is to determine how these *Q *genes are expressed differently under two conditions. To answer this question, we consider the expression values of the *Q *genes for a particular sample under a particular condition as a *Q*-dimension vector. Then the expression values of a pathway under one condition *j *can be modeled as set *S*_*j *_(*j *= 1, 2) of points in a *Q*-dimension space. The idea is to consider the two sets of points *S*_1 _and *S*_2 _as samples from two different fuzzy sets. We then examine the membership value of each element with respect to these two fuzzy sets and determine the *d*-value between the two sets of samples.

The mean ${\vec{\mu }}_{j}$ of the expression values of set *S*_*j *_is:

(1) $${\vec{\mu }}_{j}=\frac{1}{N_{j}}\sum_{{\vec{x}}_{n}\in S_{j}}{\vec{x}}_{n}$$

where,

$${\vec{\mu }}_{j}=\left[\begin{array}{l} \mu_{j1}\\ \mu_{j2}\\ \ldots \\ \mu_{jQ} \end{array}\right]\text{ and }{\vec{x}}_{n}=\left[\begin{array}{l} x_{n1}\\ x_{n2}\\ \ldots \\ x_{nQ} \end{array}\right]$$

*N*_*j *_is the number of samples in *S*_*j*_, ${\vec{x}}_{n}$ is vector made by the expression values of the n-th sample under condition j.

We then characterize set *S*_*j *_(*j *= 1, 2) by a fuzzy set *FS*_*j *_(j = 1, 2) whose fuzzy membership function is defined as:

(2) $$f_{FS_{j}}(\vec{x})=\exp\left(-\frac{1}{2}{\left({\vec{x}}_{n}-{\vec{\mu }}_{j}\right)}^{T}\Sigma_{j}^{-1}({\vec{x}}_{n}-{\vec{\mu }}_{j})\right)$$

where,

(3) $$\Sigma_{j}=\frac{1}{N_{j}-1}\sum_{{\vec{x}}_{n}\in S_{j}}({\vec{x}}_{n}-{\vec{\mu }}_{j}){\left({\vec{x}}_{n}-{\vec{\mu }}_{j}\right)}^{T}$$

Given an element $\vec{e}$ in *S*_1_, we calculate its element misclassification degree with respect to *FS*_2 _as

(4) $$m(\vec{e},FS_{2})=Max(f_{FS_{2}}(\vec{e})-f_{FS_{1}}(\vec{e}),0)$$

We denote the misclassified elements in *S*_1 _with respect to *FS*_2 _as *M*_*FS2*_(*S*_1_) = {$\vec{e}$|$\vec{e}$ ∈ *S*_1 _∦ *m *($\vec{e}$, *FS*_2_) > 0}. Similarly, we denote the misclassified elements in *S*_2 _with respect to *FS*_1 _as *M*_*FS1 *_(*S*_2_) = {$\vec{f}$|∈ *S*_2 _∦ *m *($\vec{f}$, *FS*_1_) > 0}. We denote the number of misclassified elements in *S*_1 _and *S*_2 _with respect to each other as # *M *(*S*_1_, *S*_2 _= |*M*_*FS2 *_(*S*_1_)| + |*M*_*FS1 *_(*S*_2_)|. We then define the convergence degree (*c*-value) of *S*_1 _and *S*_2 _as a linear interpolation of the number of misclassified elements and the mutual misclassification degrees as follows.

(5) *c*(*S*_1_, *S*_2_) = β*T_1 _+ (1-*β*) * *T*_2_

where,

(6) $$T_{1}=\frac{\#M(S_{1},S_{2})}{S_{1}+S_{2}}$$

and

(7) $$T_{2}=\frac{\sum_{\vec{e}\in S_{1}}m(\vec{e},S_{2})+\sum_{\vec{f}\in S_{2}}m(\vec{f},S_{1}))}{S_{1}+S_{2}}$$

Then, the divergence between *S*_1 _and *S*_2 _can be calculated using the following:

(8) *d*(*S*_1_, *S*_2_) = 1-*c*(*S*_1_, *S*_2_)

In our method, to negate the effect of outliers, we used *α*-trimmed mean instead of normal mean. The *α*-trimmed mean is calculated by ordering the sample under consideration and taking away the smallest and largest *α *values from the ordered sample. The mean of the remaining values in the sample is *α*-trimmed mean of the sample. For instance, if we have a sample of (3, 17, 25, 29, 23, 53, 22, 31, 45, 81, 90, 1), the 2-trimmed mean is calculated by removing the smallest two values (1, 3), and largest two values (81, 90) from the sample set. The mean of the remaining values (30.625) becomes the 2-trimmed mean of the sample.

For computational simplicity, an *Epanechnikov *function shown as following can be used instead of the Gaussian function of equation (2):

(9) $$f_{FS_{j}}({\vec{x}}_{n})=Max\{0,1-\frac{||{\vec{x}}_{n}-{\vec{\mu }}_{j}|{|}^{2}}{\sum_{q=1}^{Q}\sigma_{q}^{2}}\}$$

where,

(10) $$\sigma_{q}=\sqrt{\frac{1}{N_{j}-1}\sum_{{\vec{x}}_{n}\in S_{j}}{\left({\vec{x}}_{n}-{\vec{\mu }}_{j}\right)}^{2}}$$

## Analysis of method

### One dimension: a special case

In this section we analyze MCM-test for it theoretical justification. For the sake of clarity, we start with one dimension, the simplest and special case of multi-dimension. The one dimensional MCM-test corresponds to differential analysis of a single gene.

**Figure 6:**
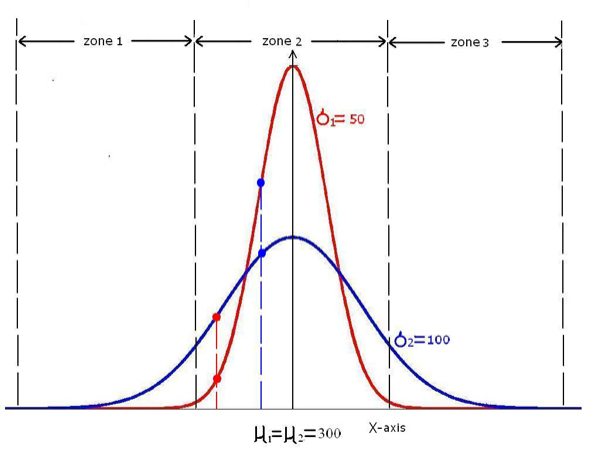
One dimension Gaussian distributions, *μ*1 = 600, *μ*2 = 700, *σ*1 = 50, *σ*2 = 100.

In Figure 6, two distributions, *D*_1 _and *D*_2 _are displayed in blue and red respectively, with mean *μ*_1 _= 600 and standard deviation *σ*_1 _= 50 for *D*_1 _and *μ*_2 _= 700, *σ*_2 _= 100 for *D*_2_. The visualization tells us that they are different as they cover different areas and have different shapes. The CM-test, which can be considered as a special case of the MCM-test differentiate them by measuring the differences on the Y axis, which is a combined result of the location difference together with the difference of the variances.

MCM-test uses the probability distribution functions of these two distributions as their fuzzy membership functions respectively, and takes advantage of the membership differences of "misclassified" samples. As shown in Figure 6, a sample *x*_1 _of *D*_2 _has a higher degree of belonging to *D*_1_, thus is "misclassified" in MCM-test. This misclassification degree is aggregated with all the other "misclassified" samples of *D*_2 _that are misclassified. Similarly, *x*_2 _of *D*_1 _has a higher degree for *D*_2_, thus is also misclassified. This misclassification degree is also aggregated with all the other misclassified samples of *D*_1_.

MCM-test collects all the misclassified degrees and the number of misclassified samples and form them into an index to measure the divergence of these two distributes. With the mean difference between these two distributions increases, the number of misclassified samples, as well as the aggregated misclassification degree decreases. Thus the MCM *d*-value will decrease.

### Two and higher dimensions

**Figure 7:**
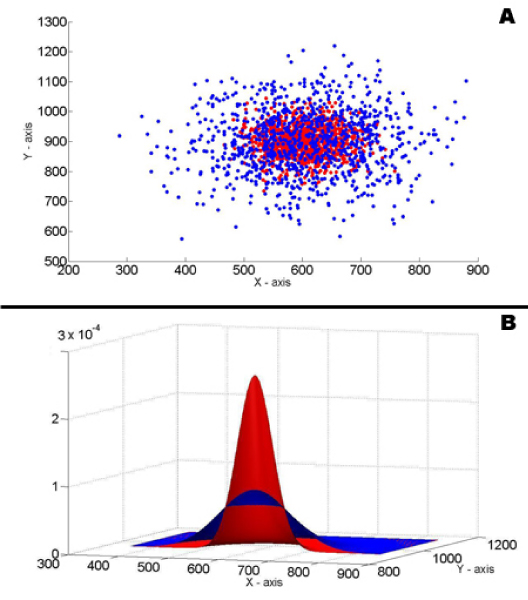
(a) 1000 samples of 2-D Gaussian distribution *μ*_1_x = 600, *μ*_1_y = 900, *σ*_1_x = *σ*_1_y = 50 and 1000 samples of 2-D Gaussian distribution *μ*_2_x = 700, *μ*_2_y = 1000, *σ*_2_x = *σ*_2_y = 100. (b) Probability density functions of the two distributions.

Figure 7(a) illustrates samples of two distributions, each of which is a 2-D Gaussian function. In pathway analysis, the *X *and *Y *axis can be the expression data of two individual genes respectively. Figure 7(b) shows the probability density functions of these two distributions, which can be used as their fuzzy membership functions after multiplying a constant.

Distributions of higher dimensions are hard to visualize. But the idea of the misclassification test stays the same. In multi-dimension space, each sample is a vector. And their misclassification degrees are used to measure the divergence of their distributions.

## List of abbreviations

MCM-test: multi-dimensional Cluster Misclassification test

CM-test: cluster misclassification test

FM-test: fuzzy membership test

GSEA: gene set enrichment analysis

## Competing interests

The authors declare that they have no competing interests.

## Authors' contributions

LRL designed the algorithm, coordinated the project and wrote part of the manuscript. VM implemented the algorithm, conducted experiments and wrote part of the manuscript. YL designed experiments and wrote part of the manuscript. DK located gene expression and pathway data for experiments, analyzed the results and wrote part of the manuscript.
